# TGF-β1/BSA coating modulates multi-phasic scaffolds for osteochondral tissue regeneration

**DOI:** 10.1016/j.mtbio.2025.101879

**Published:** 2025-05-17

**Authors:** Farnaz Ghorbani, Behafarid Ghalandari, Rainer Detsch, Chaozong Liu, Aldo R. Boccaccini

**Affiliations:** a Institute of Biomaterials, Department of Materials Science and Engineering, University of Erlangen-Nuremberg, Cauerstrasse 6, 91058, Erlangen, Germany; b Institute of Orthopaedic & Musculoskeletal, Division of Surgery & Interventional Science, University College London, Royal National Orthopaedic Hospital, Stanmore, London, HA7 4LP, United Kingdom; c Department of Translational Health Science, Bristol Medical School, University of Bristol, Bristol, BS1 3NY, United Kingdom; d Department of Surgical Biotechnology, Division of Surgery and Interventional Science, University College London, London, NW3 2PF, United Kingdom

**Keywords:** 3D printing, Electrospinning, Protein coating, Osteochondral, Tissue engineering

## Abstract

Bioinspired scaffolds, designed to replicate distinct regions and mimic stratified anatomical architecture, have emerged as a promising approach for addressing osteochondral defects (a joint injury affecting both cartilage and underlying bone). Despite extensive preclinical research, the challenge of integrating newly formed bone and cartilage has hindered clinical adoption, driving the continuous development of more effective constructs. To address this issue, we propose an approach centred on a protein-modified stratified multi-phasic scaffolds. In this investigation, we developed a bottom layer composed of polydopamine-modified 3D printed poly (lactic-co-glycolic acid) (PLGA) scaffolds loaded with simvastatin, complemented by an upper layer consisting of electrospun PLGA-gelatine fibres obtained by a green strategy, e.g., using a benign solvent. Scaffolds were then coated with transforming growth factor-β1 (TGF-β1)- bovine serum albumin (BSA). The multi-phasic scaffolds exhibited a hierarchical interconnected porous microstructure with hydrophilicity characterized by a contact angle of 24° and a swelling rate of 467 % over 24 h (n = 5), contributing to *in-vitro* hydrolytic degradation under controllable degradation rates of 49 % over 4 weeks (n = 5). Scaffolds were also shown to undergo hydroxyapatite mineralization. The multi-phasic scaffolds exhibited a cytocompatible support for adhesion and proliferation (3.5-fold increase from day 2 to day 7) of sheep bone marrow mesenchymal stem cells along with alkaline phosphatase (ALP) secretion (1.4-fold increase from day 14 to day 21) and biomineralization (n = 5). Additionally, the expression of collagen type II (COL2A1) and SRY-Box transcription factor 9 (SOX9) biomarkers increased over 28 days of cultivating human chondrocytes. Similarly, osteopontin (SPP1) and collagen type I (COL1A1) biomarkers showed increased expression over a 28-day period following the culture of human osteoblasts. These findings demonstrate the enhanced osteogenic and chondrogenic performance of the multi-phasic scaffold, intensified by the synergistic influence of the TGF-β1/BSA complex, potentially augmenting growth factor bioavailability for cells. In conclusion, the hierarchical multi-phasic scaffolds introduced in this work represent a highly promising strategy for the regeneration of osteochondral defects.

## Graphical abstract

**Figure:**
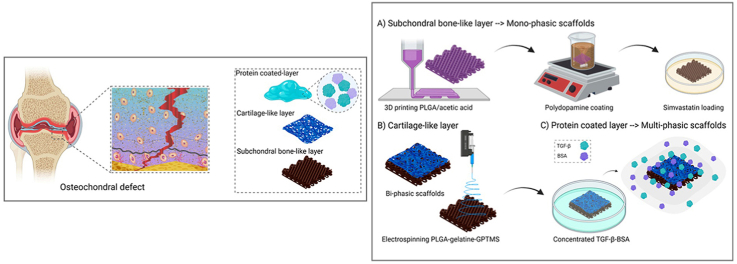

## 1 Introduction

Osteoarthritis, characterized by osteochondral defects causing damage to both the subchondral bone and cartilage [1], is a degenerative pathology that significantly impacts joint functionality, thereby diminishing an individual's overall well-being. Herein, inflammatory cytokines not only accelerate cartilage deterioration but also disrupt the osteochondral interface, with downstream effects on subchondral bone remodelling, vascular infiltration, and joint instability [[2], [3], [4]].

Osteochondral defects demonstrate a limited inherent capacity for self-repair, a phenomenon primarily attributable to the avascular nature of articular cartilage [5]. While traditional therapeutic methods, such as cell-based approaches, osteochondral transplantation, and microfracture, offer transient relief, they often yield suboptimal long-term outcomes [6]. In response to joint pathologies, tissue engineering has developed as a promising strategy for treatment. However, conventional tissue engineering scaffolds often fall short in mimicking the complex architecture of native osteochondral tissue and reproducing its topographical features aligned with anatomical fidelity [7,8]. Additionally, modulating crucial biochemical cues—such as transforming growth factor-beta 1 (TGF-β1), which plays a pivotal role in chondrogenic and osteogenic differentiation [9,10]—remains a challenge, as these signals are essential for guiding spatial and temporal cell differentiation and tissue development. The inability to deliver and localize these cues in a controlled and zonally specific manner by traditional scaffolds remains a major challenge in osteochondral tissue engineering. Addressing these limitations necessitates replicating the hierarchical structure inherent in the natural osteochondral extracellular matrix (ECM). It also requires replicating the biochemical factors required for efficient tissue regeneration. This objective can be realized through the utilization of engineered design and appropriate manufacturing techniques, which have demonstrated efficacy as a viable therapeutic approach for osteochondral defects.

The healing of osteochondral defects with zonal structure requires a novel bi/multi-phasic scaffolds design. The integration of dual fabrication techniques, involving 3D printing and electrospinning with the deposition of electrospun fibres onto a 3D printed layer, represents a promising approach in scaffolds design for osteochondral tissue regeneration, synergistically contributing to the overall structural and functional characteristics of the scaffolds. In the construction of the scaffolds, 3D printing is employed to replicate the intricate architecture of the subchondral bone layer. This method ensures not only the reproduction of the native bone structure but also provides mechanical cues for cell proliferation and differentiation [11]. Concurrently, electrospinning is employed to fabricate the top layer of the scaffolds, simulating a cartilage-like environment. The electrospun fibres create a nanofibrous network that mimics the natural ECM of cartilage, promoting cell adhesion, migration, and proliferation by introduction of a high surface area with fine-scale features [12,13]. This dual-technique scaffold design offers a harmonious combination of physicochemical properties and *in-vitro* demands, ensuring the scaffolds can be stable and it integrates with the surrounding environment during tissue regeneration.

Multi-material scaffolds have emerged as highly promising constructs, strategically combination of diverse materials to replicate the intricate biochemical properties inherent to both subchondral bone and cartilage. These scaffolds are designed to establish a conducive microenvironment, fostering crucial processes such as cell attachment, proliferation, and differentiation, thereby providing a tuneable response conducive to optimal tissue regeneration [14]. The combination of biodegradable natural and synthetic polymers, exemplified by poly (lactic-co-glycolic acid) (PLGA) and gelatine, has demonstrated remarkable versatility in affording tuneable physicochemical and *in-vitro* properties [15]. Moreover, the incorporation of polydopamine (PDA) within these scaffolds has been explained to achieve notable osteogenicity, bioactivity, and biocompatibility [16]. The synergistic combination of PDA with a bioactive pharmaceutical agent such as simvastatin emerges as an especially attractive proposition for subchondral bone and osteochondral regeneration scaffolding [17]. For rational reasons, simvastatin was selected as the active loading agent due to its well-documented osteogenic properties [17,18]. Its primary mechanism of action involves the upregulation of bone morphogenic protein-2 (BMP-2) expression. By stimulating BMP-2 expression, simvastatin promotes osteoblast differentiation, and matrix mineralization, which are essential for subchondral bone regeneration [[19], [20], [21]]. The combination of these advanced biomaterials holds substantial promise in advancing the field, paving the way for innovative solutions in osteochondral regeneration.

Surface modification of scaffolds plays an important role in augmenting the effectiveness of tissue regeneration, particularly in osteochondral tissue repair and regeneration. Employing a protein coating strategy for modifying scaffolds with bioactive signalling molecules, such as transforming TGF-β and bovine serum albumin (BSA), stands out as a promising methodology geared towards ensuring a controlled and uniform dispersion of bioactive molecules on the scaffolds. This process facilitates a spatially organized functionalization, thereby enhancing cellular recognition. The incorporation of TGF-β, a potent regulator of chondrogenesis as well as osteogenesis, is considered to foster the development of functional tissue [9,10]. Simultaneously, BSA, renowned for its biocompatibility and ability to modulate cellular responses [22], refines the scaffold's surface characteristics, thereby optimizing interactions with the surrounding tissues. This innovative approach offers a tailored and efficient mechanism to stimulate the regeneration of osteochondral tissue.

In this investigation, we engineered bioinspired stratified scaffolds for the regeneration of osteochondral defects. These zonal scaffolds comprised distinct layers: a subchondral bone-like layer at the base, fabricated from 3D printed PLGA scaffolds functionalized with PDA and loaded with simvastatin; an upper layer mimicking cartilage crafted from electrospun PLGA-gelatine fibres. The integration of 3D-printed (subchondral bone-like) and electrospun (cartilage-like) structures provide hierarchical biomimetic microenvironments conducive to cell-specific responses. Subsequently, the bi-phasic scaffolds underwent modification through the TGF-β1/BSA protein coating strategy to enhance scaffolds bioactivity and modulate cellular interactions. The scaffold design was grounded in a clear, biologically driven rationale aimed at replicating the complex zonal structure and functionality of native osteochondral tissue. While 3D printing and electrospinning have been individually and, in limited cases, sequentially combined in scaffold design, these previous efforts have generally focused on structural layering without addressing spatial biochemical functionality. Each component is selected based on its established and complementary biofunctionality relevant to either bone or cartilage repair. Although several of these agents have been previously explored in isolation, no prior study has combined these bioactive components within a hierarchically structured, multi-phasic scaffold, nor has their synergistic performance been evaluated in a layer-specific *in-vitro* model for osteochondral repair. To evaluate the potential advantages of zonal scaffolds compared to mono-phasic ones, as well as the efficacy of protein coating in regeneration of osteochondral defects, comprehensive physicochemical analyses, such as morphology observations, hydrophilicity, absorption capacity, degradation behaviour, and *in-vitro* bioactivity, were then performed. Additionally, *in-vitro* cell studies were conducted using human osteoblasts and human chondrocytes to identify the optimal structure for regenerating injured osteochondral tissue. Importantly, cells influenced by the TGF-β1/BSA complex exhibited specific biomarker expression, indicating their potential to regenerate both cartilage and subchondral bone. Our results demonstrated that the bioinspired hierarchical scaffolds, with their unique spatial design, effectively met the requirements for osteochondral tissue regeneration. Consequently, the introduction of the concept of protein-modified multi-phasic scaffolds in this study offers a promising solution to address the challenges of osteochondral tissue regeneration.

## 2 Materials and methods

### 2.1 Materials

Poly lactic-co-glycolic acid (lactide:glycolide 50:50, Mw 45,000 g/mol), gelatine from porcine skin (gel strength ∼300 g Bloom, Type A), dopamine hydrochloride (Mw 189.64 g/mol), acetic acid (glacial, ≥99.7 %), hydrochloric acid (Mw 36.46 g/mol), isopropanol (Mw 50.10 g/mol), (3-Glycidyloxypropyl)trimethoxysilane (GPTMS, Mw 236.34 g/mol), simvastatin (pharmaceutical secondary standard), transforming growth factor-β1 human (TGF-β1), bovine serum albumin (BSA, lyophilized powder), p-Nitrophenyl phosphate (p-NPP), L-ascorbic acid (Mw 176.12 g/mol), dexamethasone (suitable for cell culture, ≥97 %), L-Proline (from non-animal source, suitable for cell culture), DAPI ready-made solution (1 mg/mL), Dulbecco's Modified Eagle's Medium (DMEM, low glucose, With 1000 mg/L glucose, L-glutamine, and sodium bicarbonate), Dulbecco's Modified Eagle's Medium (DMEM, high glucose, With 4500 mg/L glucose, L-glutamine, sodium pyruvate, and sodium bicarbonate), cell counting kit 8 (WST-8), fetal bovine serum (FBS, sterile filtered, cell culture tested), and Triton™ X-100 were purchased from Sigma Co., Germany/UK. Tris buffer (Mw 121.14 g/mol, ≥99.9 %) was purchased from ROTH Co., Germany. Bradford (solution for protein determination) was purchased from PanReac AppliChem ITW Reagents Co., Germany. Osteoimage™ mineralization assay was purchased from Lonza Co., USA. Calcein (AM, cell-permeant dye) was purchased from Invitrogen™ Co., USA. penicillin streptomycin ([+] 10,000 units/mL penicillin, [+] 10,000 μg/mL streptomycin) and Dulbecco's Phosphate Buffered Saline (PBS, 1X) were purchased from Gibco Co., Germany. Osteoblast differentiation medium was purchased from Cell Applications, Inc., USA. Mouse monoclonal anti-collagen type I, Mouse monoclonal anti-SOX9 (SRY-Box Transcription Factor 9), Calcein-AM, BCA protein assay kit and Trypan blue were purchased from Thermo Fisher Scientific Co., UK. Rabbit polyclonal anti-collagen type II, Rabbit polyclonal anti-osteopontin, Goat anti-rabbit IgG AlexaFluor 647, and Goat anti-mouse TgG AlexaFlour 488 were purchased from Abcam Biotechnology Co., UK.

### 2.2 Fabrication of mono-phasic 3D printed scaffolds

The ink for 3D printing of subchondral bone-like layer was formulated using PLGA with a concentration of 20 % (W/V) in acetic acid at 40 °C overnight to ensure complete solubilization and homogeneity. The fabrication of the 3D constructs was facilitated through the utilization of computer-aided design software, complemented by the employment of an extrusion 3D printer (BioScaffolder 3.1, GeSiM, Germany). The scaffolds, featuring a square design measuring 10 × 10 mm^2^, were intricately designed in a 0/90° lay-down grid pattern with a thickness of 1 mm. Printing operations were executed using a 250 μm nozzle tip under controlled conditions of 35 ± 0.5 °C temperature and 50 KPa pressure. Following printing, the scaffolds underwent immediate freezing and subsequent air-drying, at −20 °C and then 25 °C for 24 h.

Mono-phasic scaffolds were prepared through immersion of the 3D printed constructs in a solution containing dopamine hydrochloride (2 mg/mL in 10 mM Tris buffer) with gentle stirring at ambient temperature [23]. After 24 h, the specimens underwent a thorough washing with DI-water three times. Following surface functionalization with PDA, the scaffolds were immersed in a solution of simvastatin dissolved in ethanol at a concentration of 420 mM, maintained at ambient temperature for 1 h [24]. Subsequently, the scaffolds underwent another round of washing with DI-water before air-drying at ambient temperature.

### 2.3 Fabrication of bi-phasic scaffolds

The mono-phasic scaffolds were co-joined with a layer of electrospun PLGA-gelatine fibres. For the solution preparation, PLGA and gelatine were dissolved in acetic acid at a weight ratio of 50:50, achieving a concentration of 10 % (W/V). Upon complete dissolution, GPTMS was incorporated into the polymeric solution at a weight ratio of 0.5:1 (GPTMS: gelatine) and stirred for 2 h to facilitate crosslinking. Subsequently, the monophasic scaffolds were affixed to an aluminium collector, and the hybrid solution was electrospun onto the scaffolds at a flow rate of 1 mL/h and a voltage of 10 kV, with 10 cm maintained between the needle and the collector, under conditions of 45 % humidity and a temperature of 25 to form bi-phasic constructs. Following electrospinning, all samples were air-dried overnight at ambient temperature.

### 2.4 Fabrication of multi-phasic scaffolds: protein coating surface modification

To fabricate multi-phasic scaffolds, the scaffolds underwent a surface modification process involving the application of a thin layer of TGF-β1/BSA. Specifically, a solution comprising TGF-β1 at a concentration of 10 ng/μl [9] and BSA at a concentration of 1 mg/ml [22] was prepared. The scaffolds were immersed in TGF-β1/BSA solution for 10 min at room temperature. Subsequently, the modified scaffolds underwent thorough washing with DI-water and air-dried at ambient temperature.

### 2.5 Scaffolds characterization

Microstructural examination of the scaffolds was conducted utilizing field emission scanning electron microscopy (FE-SEM, Auriga, Carl-Zeiss, Germany) at an accelerating voltage of 3 kV. Prior to imaging, samples were gold sputter-coated under vacuum (5 nm thickness) for 120 s at 45 mA to minimise charging effects and ensure surface conductivity. Furthermore, pore dimensions and fibre diameters were assessed using Image Measurement software (KLONK Image Measurement Light, Edition 11.2.0.0) based on FE-SEM micrographs.

Fourier-transform infrared spectroscopy (FTIR, IRAffinity-1S, Shimadzu, Japan) employing 40 scans across the 400-4000 cm^−1^ range with a resolution of 4 cm^−1^ was utilized to analyse chemical characterization of scaffolds.

Assessment of the constructs' hydrophilicity involved determining the water droplet contact angle through a sessile drop method at ambient temperature using a contact angle goniometer (DSA30 Expert, Krüss, Germany). A 10 μL droplet of deionised water was gently deposited onto the scaffold surface, and images were captured at 0 s (immediately after deposition) and 10 s to assess both initial and short-term dynamic wettability.

To investigate the fluid absorption capacity, scaffolds underwent immersion in 15 mL of PBS solution while being incubated in a thermoshaker (Unimax 1010 and Incubator 1000, Heidolph, Germany) at a controlled temperature of 37 ± 0.5 °C and a stirring speed of 60 rpm. The scaffolds were weighed both dry and after exposure to PBS for intervals of 3, 6, 9, and 24 h. The swelling ratio, as defined by Equation (1) [25], was determined until equilibrium in fluid uptake was attained:

(Eq. 1) $$\text{Swelling}\;\text{ratio}\;\left(\%\right)=\left[\left(W-W_{i}\right)/W_{i}\right]*100$$

where W_i_ is the dry weight of the scaffolds, and W is the wet weight of the scaffolds.

*In-vitro* degradation was studied after soaking the scaffolds in 15 mL of PBS solution while being incubated in a thermoshaker at a controlled temperature of 37 ± 0.5 °C and a stirring speed of 60 rpm for 28 days. Subsequently, the scaffolds were weighed weekly, with the PBS being replaced at each measurement time. Following air-drying, the degradation ratio was calculated according to Equation (2) [26]:

(Eq. 2) $$\left(\text{Bio}\right)\text{degradation}\;\text{ratio}\;\left(\%\right)=\left|\left[\left(W_{w}-W_{i}\right)\;/\;W_{i}\right]\right|*100$$

where W_i_ is the equilibrated swollen weight, and W_w_ is the wet weight of the scaffolds at the end of each week. The dried scaffolds were also characterized by FE-SEM and FTIR.

An *in-vitro* assessment of bioactivity spanning 28 days was conducted to assess the bioactivity levels of scaffolds. The scaffolds, measuring 10 × 10 × 1 mm^3^, were immersed in 15 mL of simulated body fluid (SBF) solution and incubated in a thermoshaker at 37 ± 0.5 °C and 60 rpm, with every other day SBF refreshment. Following the 28-day incubation period, the scaffolds underwent drying and were subsequently characterized through FE-SEM, FT-IR, and XRD techniques. X-ray diffractometry (MiniFlex 600, Rigaku, Japan) was employed within the 2θ angle range of 20–60° utilizing Cu-Kα (λ 1.5418 Å) radiation.

### 2.6 Molecular docking calculations

Molecular docking calculations were performed to examine the interaction of the BSA/TGF-β1 complex with two distinct surfaces, PDA and PLGA-gelatine. The 3D structures of BSA (PDB ID: 4F5S [27]) and TGF-β1 (PDB ID: 1KLA [28]) were obtained from the RCSB. The complexation of BSA and TGF-β1 was modelled using AlphaFold3 [29]. Docking calculations, based on previous work [22,[30], [31], [32], [33]], were conducted considering the repeated basic unit (size <3 nm) of PDA and PLGA-gelatine, extending the calculations to encompass the entire scale. To ensure thorough sampling across the large and heterogeneous protein–surface interface, the exhaustiveness parameter was set to 1000, and 20 binding modes were generated per run. For analysis, only poses within a 3 kcal/mol threshold of the top-ranked binding energy were retained. The VMD package [34] and AutoDock Tools 1.5.4 [35] were employed for input file preparation and data analysis.

### 2.7 Scaffold-cell interactions

Each of mono-phasic, bi-phasic, and multi-phasic constructs (10 × 10 × 1 mm^3^) were seeded with 10^5^ sheep bone marrow mesenchymal stem cells (sBMSCs), provided from the cell bank of Institute of Orthopaedic & Musculoskeletal Science at University College London - Royal National Orthopaedic Hospital. Prior to seeding, the scaffolds underwent 1-h UV disinfection and subsequent 1-h pre-soaking in DMEM. The objective was to investigate cell viability, proliferation, and osteogenic behaviour. The scaffolds, seeded with cells, were subjected to incubation in DMEM supplemented with 10 % (V/V) FBS and 1 % (V/V) U/ml penicillin-streptomycin, following standard culture parameters: 37 ± 0.5 °C, 5 % CO_2_, and 95 % humidity. Media were refreshed every other day and it was the standard media used in 2D cultures and 3D cultures for cell viability, cell proliferation, ALP activity and Osteoimage assays.

After culturing cells for 2- and 7-days post-seeding, cell viability was assessed using a live/dead staining technique employing Calcein-AM and Propidium Iodide (PI) solutions. Following removal of the medium, the species underwent a 1-h incubation period in a solution comprising 2 μM Calcein-AM and 4 μM PI dissolved in PBS. Subsequently, cell viability was evaluated through fluorescence microscopy (Axio Observer, Carl-Zeiss, Germany) after fixation of the cells in a fluorescent fixative solution.

Cell proliferation was assessed at 2- and 7- days distinct intervals, employing the Prestoblue assay. Upon discarding of the growth medium, the cell-cultured specimens underwent incubation in DMEM supplemented with 10 % (V/V) Prestoblue for a duration of 1 h at 37 ± 0.5 °C, 5 % CO_2_, and 95 % relative humidity. Subsequently, the absorbance at 570 nm was quantified utilizing a microplate reader (Infinite M200pro, TECAN, Switzerland).

To assess osteogenic activity, the specific alkaline phosphatase (spec. ALP) activity was measured. After culturing the cells for 14 and 21 days, they were lysed using a lysis buffer. The medium was centrifuged at 1200 rpm for 10 min to separate the supernatant. This supernatant was then incubated with an spec. ALP-mix solution containing p-NPP for 90 min to allow the enzymatic reaction to occur. The reaction was stopped by adding 1M NaOH. The ALP activity was determined by measuring the absorbance at 405 nm and 690 nm. For normalization, the spec. ALP activity was adjusted according to the total protein content, which was measured using the BCA protein assay in accordance with the manufacturer's protocol.

The final osteogenic cells differentiation is characterized by the formation of mineralized nodules, which consist of inorganic hydroxyapatite. To evaluate the hydroxyapatite biomineralization of the cells, we utilized a OsteoImage™ assay. The experimentation protocol adhered to the guidelines outlined in the Lonza kit instructions, with observations conducted 21 days post-culture. Following medium removal and washing, cell fixation took place in a fluorescent fixative solution. Subsequent to two washes with diluted wash buffer solution, the cultured cells underwent staining under dark conditions, followed by a 30-min incubation period at 37 ± 0.5 °C, 5 % CO_2_, and 95 % relative humidity. After discarding the staining reagents and three additional washes using diluted wash buffer, mineralization levels were assessed using a fluorescence microscope and quantified with a microplate reader at excitation/emission wavelengths of 492/520 nm.

The analysis of osteopontin (SPP1) and collagen type I (COL1A1) expression as osteoblastic differentiation markers, along with SRY-box transcription factor 9 (SOX-9) and collagen type II (COL2A1) as chondrogenic markers, was conducted through immunocytochemistry. Scaffold disinfection involved UV treatment prior to cell seeding. Specifically, one set of scaffolds received a seeding of 10^5^ human osteoblasts/ml following a 1-h incubation in osteoblast differentiation medium, while another set seeded with 10^5^ human chondrocytes/ml after a similar incubation in chondrogenic medium (DMEM high glucose, 10 % (V/V) FBS, 1 % (V/V) Penicillin-Streptomycin, 40 μg/ml, 100 nM dexamethasone, 200 μM ascorbic acid, 10 ng/ml TGF-β1). Human osteoblasts and chondrocytes were sourced from the Institute of Orthopaedic & Musculoskeletal Science at University College London - Royal National Orthopaedic Hospital. The seeded scaffolds underwent a 21-day incubation period in osteoblast differentiation medium (for osteoblast-seeded scaffolds) or chondrogenic medium (for chondrocyte-seeded scaffolds) at 37 ± 0.5 °C, 5 % CO_2_, and 95 % humidity, with media replacement every other day.

Subsequently, cells were subsequently fixed in 4 % paraformaldehyde for 10 min, then permeabilized using 0.1 % (V/V) Triton X-100 for 15 min, followed by blocking with a 2 % (W/V) BSA solution at ambient temperature. Primary antibodies (Mouse monoclonal anti-collagen type I (1:100), Rabbit polyclonal anti-osteopontin (1:200), Rabbit polyclonal anti-collagen type II (1:200), Mouse monoclonal anti-Sox-9 (1:500)) diluted in 0.1 % (W/V) BSA were applied overnight at 4 °C, followed by staining using secondary antibodies (Goat anti-rabbit IgG AlexaFluor 647 (1:1000), and Goat anti-mouse IgG AlexaFlour 488 (1:1000)) diluted in 0.1 % (W/V) BSA for 45 min at ambient temperature. Thereafter, DAPI staining (1:1000) was performed for 5 min at ambient temperature. Cells were observed using confocal laser scanning microscopy (LEICA STP8000, LEICA, Germany).

### 2.8 Statistical analysis

Each experiment was conducted five times (n = 5), and the results were presented as the mean ± standard deviation. One-way ANOVA and two-way ANOVA followed by Tukey's post hoc test were used to compare multiple groups. For comparisons between two groups, t-tests were applied where appropriate. Normality of the data was assessed using the Shapiro–Wilk test. The statistical significance of the mean values was determined using GraphPad Prism (version 10.2.3, San Diego, California), with a significance threshold set at p ≤ 0.05 (∗P ≤ 0.05; ∗∗P < 0.01; ∗∗∗P < 0.001).

## 3 Results and discussion

### 3.1 Microstructural evaluation

**Fig. 1:**
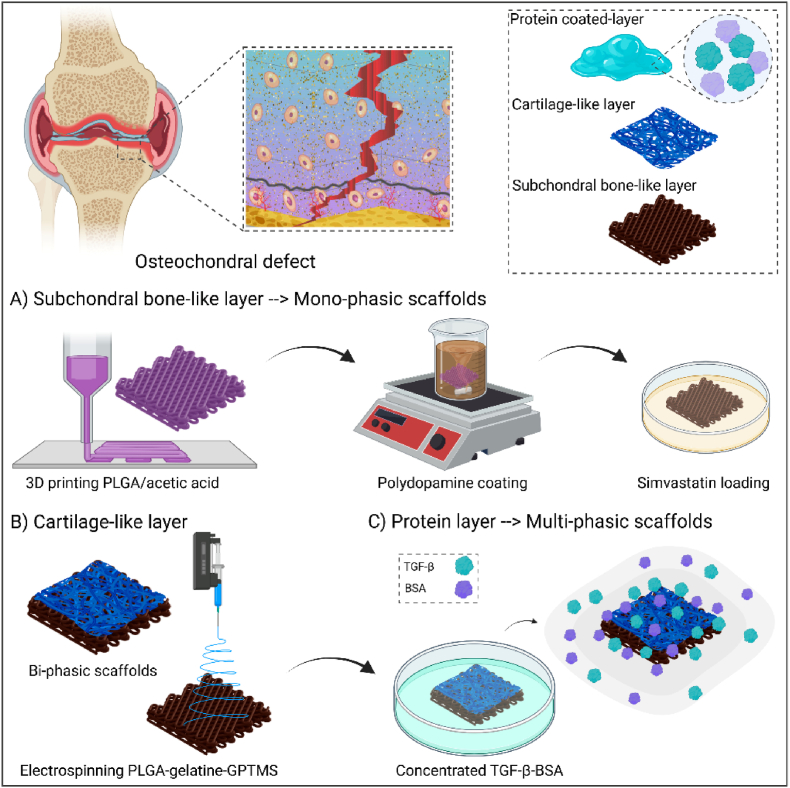
Schematic illustration of a multifaceted approach involving the integration of materials and bioactive agents to fabricate multi-phasic scaffolds for osteochondral tissue regeneration (created by www.biorender.com).

**Fig. 2:**
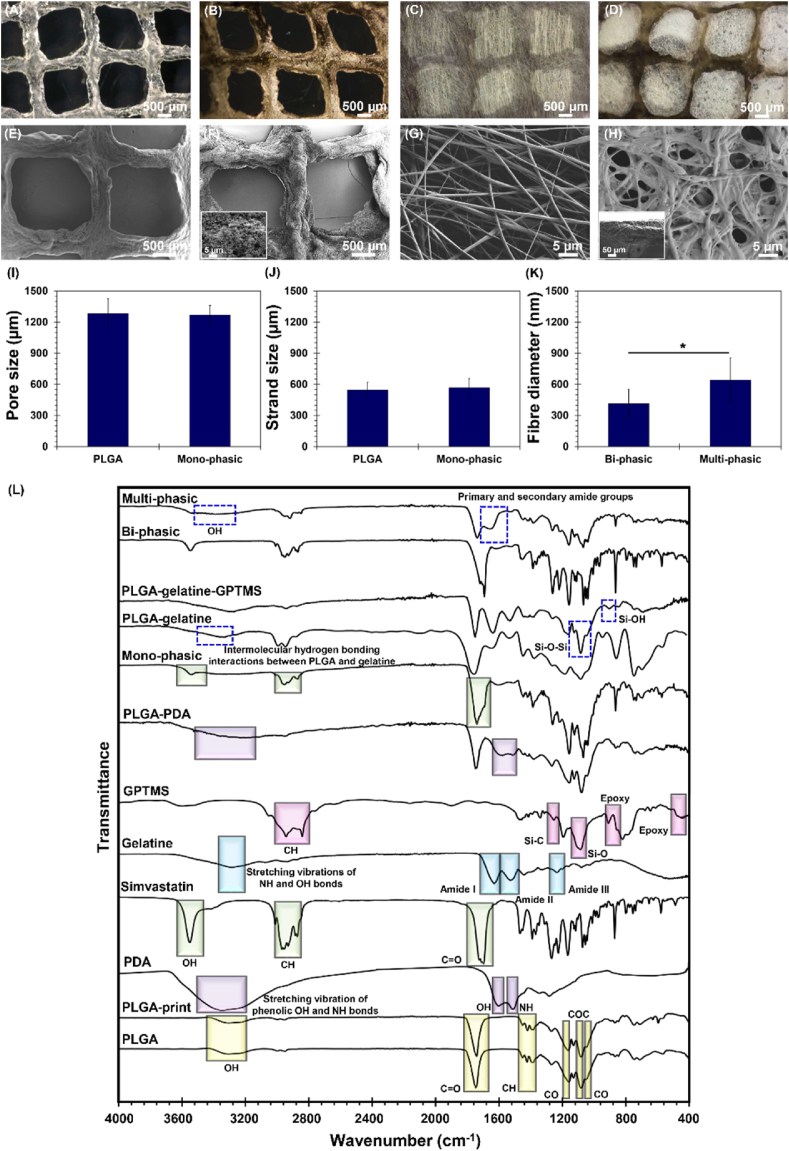
Microstructural and chemical evaluation of mono-phasic, bi-phasic, and multi-phasic scaffolds. (A–D) optical microscopy images and (E–H) FE-SEM images of (A, E) PLGA scaffolds, (B, F) mono-phasic scaffolds along with higher magnification showing functionalization with PDA (smaller image in figure F), (C, G) bi-phasic scaffolds, and (D, H) multi-phasic scaffolds along with a longitudinal section image (smaller image in figure H). (I) Average pore size, (J) average strand size of 3D printed layers, and (K) average fibre diameter of electrospun layer before and after protein coating. (∗P ≤ 0.05) (L) FTIR spectra of raw materials and 3D-printed scaffolds, confirming the presence of key functional groups and successful material integration. Main functional groups are highlighted with coloured indicators.

The architecture and surface characteristics of scaffolds profoundly influence cellular behaviour, nutrient diffusion, and integration with host tissue [36]. Accordingly, a well-designed scaffold with tailored microstructure holds promise for enhancing tissue regeneration and improving clinical outcomes. Additionally, the chemical composition of the surface influences cellular adhesion, protein interactions, and bioactive signalling. Through meticulous adjustment, such as functional groups or bioactive coatings, surface chemistry directs cellular responses, promotes tissue integration, and enables controlled drug delivery, enhancing scaffolds performance and therapeutic outcomes [30]. The aim of this study was to engineer a hierarchical scaffold tailored for osteochondral defect regeneration. This scaffold design, depicted schematically in Fig. 1, entails a multifaceted approach involving the integration of materials and bioactive agents. The bi-phasic scaffolds consist of a fibrous layer made of PLGA-gelatine connected to a lower layer made of PDA-functionalized 3D printed PLGA that contains simvastatin, providing a diverse platform for osteochondral tissue engineering applications. Additionally, the protein coating strategy has been employed to coat the scaffolds with TGF-β1/BSA complex and fabricate multi-phasic constructs, further enhancing the cytocompatibility and bioactivity, thereby augmenting the potential for translation. The morphology of the mono-phasic, bi-phasic, and multi-phasic scaffolds, as depicted in Fig. 2(A–H), underscores the intricate design, crucial for osteochondral tissue regeneration.

The microscopy analysis has unveiled a tubule-like microstructure embedded within the 3D printed PLGA layer (Fig. 2(A–E)). This architectural configuration exhibits an engineered internal structure characterized by cubic pores. Such a design fosters optimal cell infiltration and nutrient diffusion across the scaffolds matrix, consequently augmenting cell enrichment and promoting gene and protein expression, in alignment with prior research [37,38]. To enhance surface properties and induce osteogenesis potential, surface modification through spontaneous polymerization of dopamine hydrochloride has resulted in a homogeneous coating of PDA across the surface of the PLGA (Fig. 2(B–F)), as indicated by its distinct dark brown colouration. This coating provides supplementary anchorage sites for cellular adhesion, as indicated in other investigation [39]. The inherent adhesive properties of PDA, attributed to its catechol and amine functional groups, establish a conducive environment for the binding of bioactive molecules, thus facilitating cellular performance [40]. Therefore, PDA acts as a robust anchoring mechanism for the subsequent loading of simvastatin, playing a pivotal role in the controlled and targeted delivery of the pharmaceutical agent [41,42]. It is anticipated that the synergistic interplay between PDA functionalization and simvastatin loading holds significant promise for fostering enhanced therapeutic outcomes in subchondral bone regeneration.

The electrospinning technique was employed to deposit PLGA-gelatine fibres onto 3D printed scaffolds, designed to mimic the cartilage layer. Microscopic analysis of the resultant scaffolds, as depicted in Fig. 2(C–G), revealed the formation of uniform, bead-free PLGA-gelatine fibres. The coating of TGF-β1/BSA complex onto the bi-phasic scaffolds Fig. 2(D–H) was confirmed through microscopic imaging. Analysis of the FE-SEM images revealed homogeneous distribution of the TGF-β1/BSA coating across the surface, which may positively impact the adhesion, proliferation, and differentiation of osteoblasts and chondrocytes by altering the surface chemistry and topography [43]. Subsequent to the protein coating, noticeable alterations in fibre morphology were evident, which can be attributed to conformational adjustments within protein molecular structure [44] and the increased swelling induced by protein adsorption, and finally structural deformations [45]. Furthermore, cross-sectional images (Fig. 2(H)) revealed a good interconnection between the fibres and the 3D printed layer, resulting in the development of biphasic scaffolds with a cartilage-like layer thickness of ⁓100 μm. Specifically, the electrospun nanofibres were deposited directly onto the 3D-printed structure, allowing intimate contact during solvent evaporation. Importantly, the chemical similarity of the base polymers (PLGA in both layers) and the compatibility of solvents used during electrospinning and scaffold preparation are expected to enable interfacial entanglement and fusion, facilitating cohesive bonding at the material interface. Similar results were observed by Lee et al. [46]. Remarkably, other studies indicated that the integration of electrospun fibres onto 3D printed scaffolds lead to enhancement in stem cell adhesion and proliferation, surpassing that of unmodified 3D printed scaffolds [47]. Consequently, the conceptualization of stratified scaffolds can present a promising avenue for bolstering cellular activities and facilitating the regeneration of osteochondral tissues.

The mean pore diameter and strand width (Fig. 2(I and J)) of 3D printed PLGA scaffolds were measured to be 1283 ± 142 μm and 546 ± 74 μm, respectively, prior to coating. Following coating with PDA and loading simvastatin, these dimensions remained largely unchanged, suggesting the robustness of the original scaffolds’ architecture. However, an alteration in pore diameter and strand width to 1269 ± 92 μm and 569 ± 87 μm, respectively, was observed post-functionalization, which may suggest enhanced stability facilitated by the deposition of PDA and simvastatin. Moreover, the electrospun fibres exhibited an average diameter of 415 ± 137 nm, as indicated in Fig. 2(K). Remarkably, upon coating with proteins, a significant increase in fibre diameter to 641 ± 213 nm was noted, implying fibre fusion and reduced inter-fibre spacing. This phenomenon is anticipated to reinforce scaffolds integrity and enhance cell adhesion due to increased contact area.

The dimensions of the pores can closely mirror the architecture of natural bone or cartilage matrices and play a pivotal role in fostering cellular infiltration and tissue development within scaffolds, thereby offering enhanced support for osteogenesis or chondrogenesis [48]. For bone repair, pore sizes typically range from 100 to 900 μm [[49], [50], [51]], whereas for cartilage repair, dimensions in the range of 200–500 μm are preferred [52]. Notably, recent studies by Wang et al. [53] have highlighted the significance of larger pore diameters, such as 1000 μm, in promoting stem cell adherence, growth, and osteogenesis, consequently enhancing bone regeneration and vascularization. In the context of this study, the 3D printed layers were characterized by relatively large pore sizes, a feature anticipated to facilitate cell adhesion and differentiation, albeit potentially requiring extended time for cells to populate and fill the centre of defect [54,55]. However, the incorporation of electrospun fibres atop the 3D printed scaffolds form a dense network on the surface, serving as anchorage sites accessible to cells while simultaneously imposing a physical barrier that reduces the large pore size available for cell infiltration and tissue ingrowth [56]. Moreover, the presence of electrospun fibres enhances the available surface area for cell attachment and interaction, potentially amplifying cellular responses within the scaffolds. Additionally, the integration of electrospun fibres contributes to the formation of a graded pore structure in the entire scaffold, characterized by smaller pores at the scaffolds surface transitioning to larger pores deeper within the construct. This gradient mimics the natural ECM, thereby facilitating cell migration and tissue integration throughout the scaffolds [1,57].

### 3.2 Chemical characterization

The FTIR analysis presented in Fig. 2(L) elucidates distinctive spectrum of both individual constituents and multi-material constructs. Pure PLGA exhibits distinctive absorption bands indicative of its structure. Notably, the stretching vibration of the C=O bond is discernible at approximately 1750 cm^−1^, alongside C-O peaks at 1150 cm^−1^ and 1050 cm^−1^. A broad peak observed between 3200 and 3400 cm^−1^ signifies the presence of hydroxyl groups within the PLGA. Furthermore, FTIR reveals the presence of characteristic peaks corresponding to the CH bond at 1380–1430 cm^−1^ and stretching of the ether group at 1080 cm^−1^ [58,59]. Following the dissolution of PLGA in acetic acid and subsequent fabrication of 3D printed scaffolds, spectral analysis indicates the complete elimination of acetic acid, with no discernible peaks associated with its presence. This observation alleviates concerns regarding the acidic component and its potentially harmful effects on cells. Here, through the utilization of an environmentally friendly solvent, we aimed to not only achieve effective scaffolds fabrication but also to align with sustainability goals in materials science.

Synthesized PDA showcases unique OH and NH peaks, discernible around 1603 cm^−1^ and 1513 cm^−1^, respectively. The broad peak in the range of 3200–3500 cm^−1^ signifies the stretching vibration of phenolic OH and NH bonds found in the catechol units of PDA [60]. Notably, the peaks associated with PDA on PLGA-PDA scaffolds validate the spontaneous oxidation process of dopamine hydrochloride on the PLGA matrices. The attachment of PDA to the PLGA may occur through diverse binding mechanisms [61]. PDA contains catechol groups capable of undergoing Michael addition reactions with carbonyl groups within PLGA, thereby facilitating the formation of covalent bonds. Additionally, non-covalent interactions, including hydrogen bonding, may arise between the hydroxyl or carboxyl functional groups of PLGA and hydroxyl or amine groups of PDA.

The simvastatin spectrum reveals characteristic peaks, including vibrations of the C–H group at 2870–2970 cm^−1^, as well as the C=O and ester group vibrations at 1700 cm^−1^. Moreover, the presence of O–H vibrations in approximately 3500 cm^−1^ indicates the involvement of hydroxyl functional groups within the simvastatin molecule [62]. Simvastatin may physically interact with the PDA-functionalized matrix, potentially involving processes such as adsorption.

The FTIR analysis of gelatine revealed the peaks located at 1631 cm^−1^, 1521 cm^−1^, and 1230 cm^−1^ corresponded to the amide I, amide II, and amide III bands, respectively [63]. Additionally, the peak at 3200–3400 cm^−1^ indicated the stretching vibrations of N-H and O-H bonds [64]. Following the incorporation of gelatine into the PLGA matrix, amide peaks appeared in composite spectrum. Furthermore, the spectral region around 3400 cm^−1^ provides insight into the intermolecular hydrogen bonding interactions between PLGA and gelatine [65].

The chemical analysis of GPTMS indicated peaks at 2944 and 2812 cm^−1^, attributable to the CH_3_ stretching vibrations inherent to the methoxy groups within the GPTMS structure. Furthermore, distinct peaks at 1080 cm^−1^ and 1255 cm^−1^ were observed, indicative of the Si-O and Si-C, respectively. Additionally, peaks at 914, 849, and 436 cm^−1^ were identified in the FTIR spectrum, confirming the presence of epoxy groups within the GPTMS structure [66,67]. Comparison between gelatine and composite fibres elucidated peaks indicative of successful cross-linking between gelatine and GPTMS, notably the appearance of Si-O-Si bands at 1035 cm^−1^ and 1085 cm^−1^, as well as Si-OH bands at 900 cm^−1^ [68,69].

The aforementioned peaks were identified within bi-phasic and multi-phasic scaffolds. Notably, within multi-phasic scaffolds, discernible peaks, particularly at approximately 3400 cm^−1^ and 1650 cm^−1^, signify the presence of OH stretching and primary and secondary amide groups inherent to BSA [70]. Furthermore, spectral features within the range of 1600–1700 cm^−1^ correspond to the structural motifs of α-helix, β-sheet, and β-turn characteristic of TGF-β1 [71]. These features become prominently evident subsequent to the application of the protein complex onto the bi-phasic scaffolds surface. Upon immersion of the scaffolds in a TGF-β1/BSA solution, it is possible that the protein complex undergoes structural changes to enhance its interaction with the substrate. Such adaptations may entail conformational alterations and the reorientation of protein domains, which are influenced by the thermodynamics and kinetics of the system involved [72,73], potentially leading to more area to interact with surface functional groups such as carboxyl, amino, and hydroxyl moieties, present within the upper and lower layers of the scaffolds. This effect potentially contributes to the structural integrity and stability of the protein-scaffold complex. The formation of Schiff base interactions is plausible, facilitated by the nucleophilic attack of amine groups from proteins onto the carbonyl groups inherent in the scaffolds, leading to the establishment of covalent bonds [74]. The hydroxyl, carboxyl, and amide functional groups inherent to proteins possess the capacity to engage in hydrogen bonding interactions with hydroxyl functional groups within the chemical structure of the scaffolds, or alternatively, they can partake in electrostatic interactions with charged functional groups present on the surface of the scaffolds [75].

### *3.3* *In-vitro* absorption capacity and degradation

**Fig. 3:**
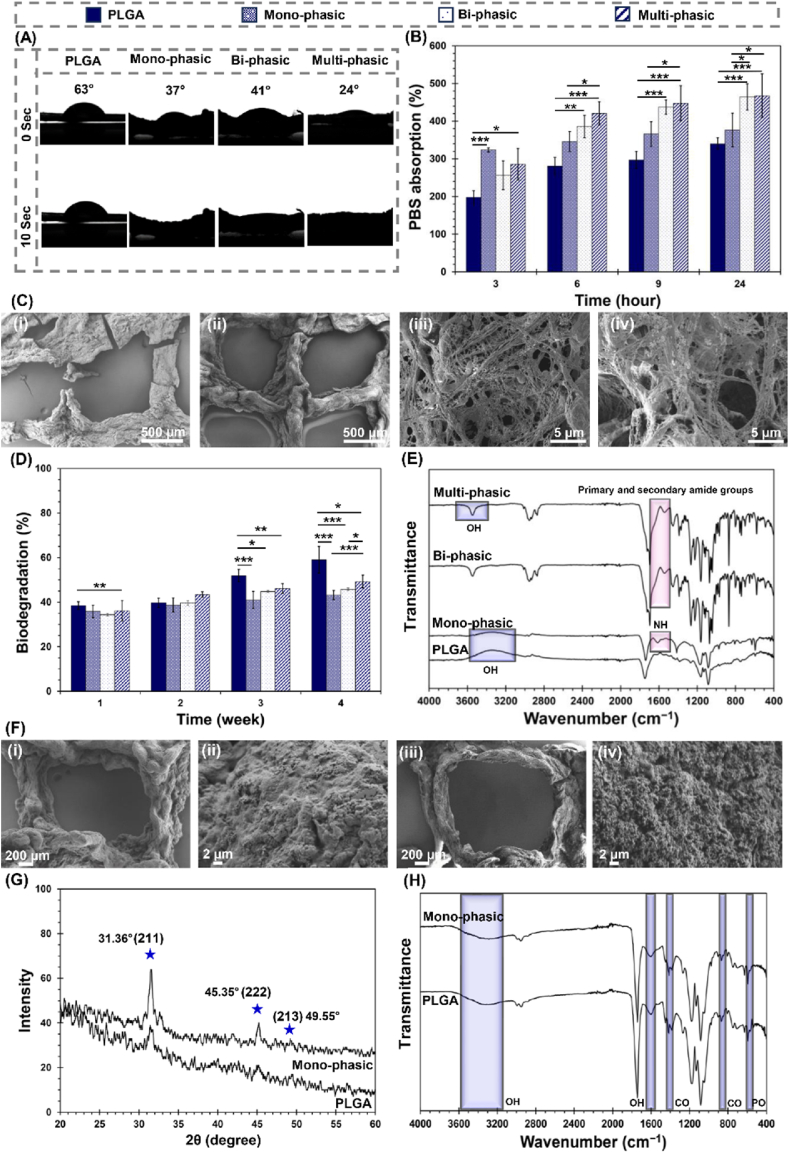
Interaction of scaffolds with biological-like fluids. (A) Hydrophilicity of the PLGA, mono-phasic, bi-phasic, and multi-phasic scaffolds using water-drop contact angle method. (B) PBS absorption ratio of scaffolds over time (3, 6, 9, and 96 h), (∗P ≤ 0.05; ∗∗P < 0.01; ∗∗∗P < 0.001). (C–E) *In-vitro* degradation characterization of scaffolds, (C) FE-SEM micrographs of (i) PLGA and (ii) mono-phasic, (iii) bi-phasic, and (iv) multi-phasic degraded scaffolds after 28 days of immersion in the PBS solution, (D) Degradation ratio of scaffolds over time (1, 2, 3, and 4 weeks), (∗P ≤ 0.05; ∗∗P < 0.01; ∗∗∗P < 0.001), (E) FTIR spectra of degraded scaffolds after 28 days of immersion in the PBS solution. (F–H) *In-vitro* mineralization of the scaffolds, (F) FE-SEM micrographs of (i, ii) PLGA and (iii, iv) mono-phasic scaffolds after 28 days of immersion in the SBF solution, (G) XRD pattern and (H) FTIR spectra and of scaffolds after biomineralization of HA-like layers.

Water-scaffold interactions facilitate the transport of nutrients and waste within the scaffolds, aiding in cell growth, migration, and differentiation, thus affecting vascularization and tissue formation [76]. Contact angle measurements were conducted to evaluate the wettability of the scaffold surfaces. Contact angle measurements on porous or fibrous scaffolds may be affected by capillary absorption and surface topography, which can lead to deviations from the idealised values typically observed on flat, homogeneous films. Nevertheless, our aim was to assess the effective wettability of the scaffolds in their functional 3D architecture, rather than isolating surface chemistry alone. Given that cell–scaffold interactions in tissue engineering occur at the real, three-dimensional interface, evaluating wettability under these conditions offers practical relevance for predicting biological responses and enables a meaningful comparison between different scaffold formulations. According to the wettability assessment, the PLGA, mono-phasic, bi-phasic, and multi-phasic scaffolds demonstrated contact angles of 63°, 37°, and 41°, and 24° respectively, as illustrated in Fig. 3(A). The measured contact angle of 24° in multi-phasic scaffolds confirms the high hydrophilicity of the scaffold surface, a crucial factor influencing cell adhesion and bioactivity. Studies have shown that surfaces with a contact angle in the range of 20–40° promote optimal integrin-mediated adhesion, leading to improved initial cell attachment and subsequent cellular responses [77]. Interestingly, while the PLGA group maintained consistent wettability over time, the other experimental groups exhibited time-dependent changes. Notably, after 10 s, the water drop was absorbed, particularly in the mono-phasic and multi-phasic scaffolds. This absorption was attributed to the presence of specific surface modifications, such as PDA in the mono-phasic scaffolds and protein complexes in the multi-phasic scaffolds.

**Fig. 4:**
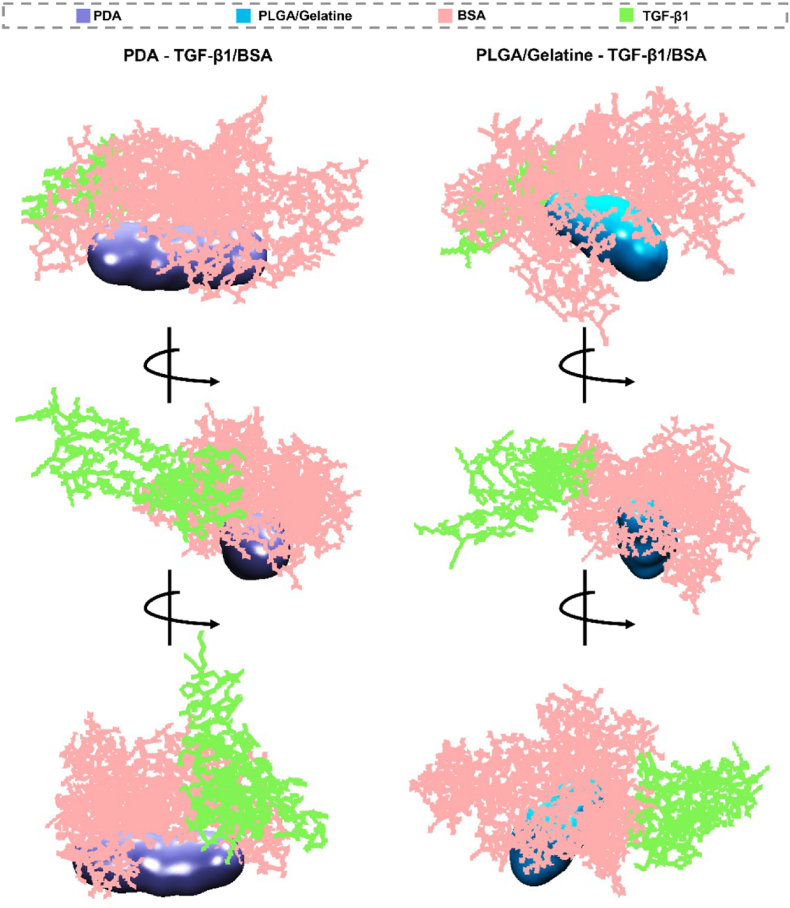
Protein-polymer interactions. TGF-β1/BSA complex binding to the surfaces of (A) PDA and (B) PLGA-gelatine.

The investigation into the absorption capacity of scaffolds revealed compelling dynamics over time, as detailed in Fig. 4(B). Initially, a notable PBS uptake occurred across all scaffolds within the initial 3-h period, attributable to the interconnected porous microstructure and the presence of hydrophilic functional groups inherent in the scaffold's composition, facilitating rapid PBS infiltration. As a function of time, absorption rates exhibited a consistent enhancement, culminating in equilibrium swelling after 24 h. Of particular significance is the achievement of equilibrium absorption ratios for PLGA scaffolds, reaching at 340 ± 16 % after this duration. This value reached to 377 ± 45 % in mono-phasic scaffolds. The observed hydrophilicity and absorption capacity within mono-phasic matrices can be ascribed to the enrichment of surface functionalities with catecholamine and hydroxyl groups. This alteration in surface chemistry promote robust hydrogen bonding with water molecules, thus manifesting heightened hydrophilic behaviour [78]. Noteworthy is the impact of PLGA-gelatine fibre integration on this hydrophilicity, discernible from the equilibrium absorption ratios of bi-phasic scaffolds (464 ± 35 %). Interestingly, the initially lower wettability and absorption capacity observed in bi-phasic scaffolds compared to mono-phasic constructs can be attributed to the presence of hydrophobic GPTMS in their chemical structure. A similar effect of GPTMS was reported by Wang et al. [79]. However, over time, this effect diminished, and the absorption potential of bi-phasic scaffolds increased. This phenomenon can be explained by the hydrophilic gelatine within the fibres becoming more exposed and accessible, leading to an increased affinity for water molecules. Additionally, the dynamic nature of scaffolds hydration and swelling processes allows for the rearrangement and reorientation of polymer chains and surface functionalities, leading to the creation of more hydrophilic pathways within the scaffolds structure, promoting improved fluid infiltration and absorption over time. Furthermore, the application of TGF-β1/BSA coating resulted in equilibrium absorption ratios of 467 ± 58 % in multi-phasic scaffolds post 24 h of incubation. This implies that TGF-β1/BSA complex introduces hydrophilic characteristics to the scaffolds, facilitated by the presence of polar amino acid residues. These hydrophilic functional groups also foster robust hydrogen bonding with water molecules, thereby facilitating PBS absorption. The substantial improvement in absorption ratio underscores the potential of TGF-β1/BSA coating in tailoring scaffolds properties.

The biodegradability of scaffolds stands as a pivotal characteristic dictating their performance and clinical relevance. The gradual degradation of scaffolds over time aligns with the pace of tissue remodelling, ultimately leaving behind regenerated tissue without the need for surgical intervention. This controlled degradation process not only facilitates seamless integration with host tissue but also mitigates adverse inflammatory responses, fostering a conducive environment for cell proliferation, differentiation, and ultimately functional tissue restoration [80]. The degradability assessment of hierarchical scaffolds was conducted using PBS solution. Examination of the FE-SEM images following the four-week degradation test (Fig. 3(C)) revealed the emergence of crack formations within the printed PLGA strands, suggesting a gradual degradation process occurring within the scaffolds structure. Functionalization of scaffolds with PDA demonstrated a notable enhancement in the stability of the constructs. This augmentation in stability can be attributed to the adhesive and cohesive properties of PDA, which potentially reinforce the structural integrity of the scaffolds, as discussed in other literature [81]. In addition, the application of TGF-β1/BSA coating onto the scaffolds, rendering them multi-phasic, exhibited a heightened breakdown of fibres compared to their uncoated scaffolds, which were bi-phasic. This underscores the influence of proteins hydrophilic functional groups on the surface and increased surface area on degradation kinetic.

Fig. 3(D) presents an analysis of the degradation ratio relative to polymer weight over a 4-week incubation period. The stable increase in degradation ratio observed across all scaffold types underscores the ongoing degradation processes during the incubation period. Notably, the PLGA scaffolds exhibited the highest degradation ratio among the experimental groups, with a 4-week degradation value of 59 ± 6 %. This observation may come from the inherent characteristics of PLGA, known for its susceptibility to hydrolysis processes. However, the incorporation of PDA coating on the scaffolds surface demonstrated a mitigating effect on degradation, as evidenced by reduced degradation ratios (4-week degradation of 43 ± 2 %) compared to uncoated constructs. This phenomenon may be attributed to the reinforcement effect of PDA, as observed in former investigations [81,82]. Furthermore, the bi-phasic reached the 4-week degradation of 46 ± 1 %, indicating no significant influence of the electrospun layer on the degradation of the mono-phasic scaffolds. However, multi-phasic scaffolds (4-week degradation of 49 ± 3 %) exhibited slightly higher degradation ratios compared to mono-phasic scaffolds, indicating a potential correlation between scaffolds composition and degradation kinetics. The enhanced degradation observed in the multi-phasic scaffolds is likely associated with their increased absorption capacity, likely stemming from the presence of protein coating layers and hydrophilic functional groups. While multi-phasic scaffolds exhibited a higher rate of degradation in comparison to mono-phasic and bi-phasic scaffolds, their degradation remained below 50 % within a month. This observation suggests that the degradation kinetics of these scaffolds appears to be align with the rate of osteochondral tissue formation. Osteochondral tissue regeneration is process taking over a temporal span ranging from 2 weeks to 6 months. Initially, injury triggers an inflammatory response, recruiting immune cells to remove debris and initiate repair. Stem cells derived from bone marrow migrate towards the injury site, where they undergo differentiation into chondrocytes and osteoblasts, forming new cartilage and bone. Subsequently, the neo-tissue undergoes a phase of remodelling, continuous resorption and bone formation facilitated by osteoclasts and osteoblasts, respectively. This process dynamically modulates the tissue's structure and composition to enhance its ability to withstand mechanical stress [83]. Following this, the process of cartilage tissue development begins with the cloning of chondrocytes. Over the course of about six months, the newly formed cartilage tissue undergoes gradual maturation and effectively blends and seamlessly integrates with the surrounding tissue [84]. The coordination of these regenerative processes is tightly regulated by signalling molecules such as TGF-β [9,10], which play a pivotal role in modulating cellular behaviour and matrix synthesis. Accordingly, it is anticipated that multi-phasic scaffolds enriched with TGF-β1/BSA complex will demonstrate heightened efficacy in fostering the regeneration of defective osteochondral tissue.

The investigation of structural alterations during scaffolds degradation was further elucidated through FTIR spectroscopy, complementing the findings from FE-SEM analysis and degradation kinetics analysis. Fig. 3(E) presents the FTIR spectra capturing distinctive changes in molecular composition over the degradation period. Upon incubation in PBS solution for 4 weeks, notable changes were observed in both PLGA and mono-phasic scaffolds. The disappearance of the OH peak in the spectral region of 3200–3400 cm^−1^ suggests a degradation-induced breakdown of hydroxyl groups within the scaffolds’ matrices. Additionally, a reduction in the intensity of the NH-related peak at 1513 cm^−1^, attributed to PDA, was evident in mono-phasic constructs. Furthermore, in the mono-phasic scaffolds incorporating simvastatin, the disappearance of C=O peaks corresponding to ester groups at 1700 cm^−1^ further confirms the degradation-mediated alterations. Distinct changes were also observed in bi-phasic and multi-phasic constructs. Specifically, variations in the peaks associated with primary and secondary amides at around 1600 cm^−1^ indicate degradation in the protein-based components of these scaffolds. Moreover, in multi-phasic samples featuring protein coatings, the broad OH peak turned to a tiny peak, suggesting substantial modifications in the molecular composition induced by degradation processes. These findings collectively underscore the degradability of the scaffolds in during the regeneration process.

### 3.4 Acellular mineralization

Mineralization of scaffolds enhances their resemblance to natural subchondral bone, promoting better integration. This process also fosters cell attachment and proliferation, crucial for tissue regeneration [85]. The immersion of scaffolds in SBF induces a series of chemical reactions crucial for the formation of mineralized layers. When scaffolds are placed in the SBF solution, they release hydrogen ions, which then cause hydrogen carbonate and hydrogen phosphate to be deposited onto the scaffolds [60]. These compounds serve as nucleation sites, promoting the subsequent interactions between ions present in the SBF and the scaffolds, which drives the growth of a crystalline hydroxyapatite layer. Examination of the scaffolds through FE-SEM imaging (Fig. 3(F)) corroborated the occurrence of mineralized layer precipitation. Notably, within the PLGA scaffolds, there was a little precipitation, contrasting with the observation of an aggregated calcium phosphate layer on the surface of mono-phasic scaffolds. This phenomenon is likely attributable to the influence and chemistry of PDA and simvastatin on mineralized layer formation [86].

The XRD analysis conducted on the mineralized scaffolds (Fig. 3(G)) distinctly indicates the formation of hydroxyapatite, substantiated by the presence of characteristic peaks at 2θ angles of 31.63°, 45.35°, and 49.55° corresponding to crystallographic planes (211), (222), and (213), respectively [78,87]. Such discernible peaks validate the mineralization of hydroxyapatite crystals, mirroring the crystalline structure inherent to natural bone composition. In Fig. 3(H), the FTIR spectra following bioactivity analysis are presented. An evident peak at 590 cm^−1^ signifies the presence P-O bonds within the precipitated layer. Furthermore, the presence of O-H stretching vibration is discerned through a distinct peak at 1604 cm^−1^. The emergence of C-O vibrations, denoted by peaks at 808 cm^−1^ and 1413 cm^−1^, signifies the formation of carbonated hydroxyapatite [88]. Notably, the composition of the mineralized layer closely resembles that of physiological hydroxyapatite, highlighting its potential for biomimetic applications and improved tissue integration and regeneration [89]. The FTIR spectra and XRD pattern analyses elucidate the role of PDA and simvastatin within monophasic-phasic scaffolds in fostering the nucleation and subsequent growth of hydroxyapatite crystals. This claim is supported by a noticeable increase in the intensity of peaks characteristic of mineralized hydroxyapatite. One likely reason for this phenomenon is rooted in providing functional groups, such as catechol and amine groups, by PDA that facilitate calcium ion binding, thereby promoting HA crystal formation [90].

### 3.5 Protein interaction

The molecular docking studies reveal distinct binding behaviours of the TGF-β1/BSA complex on PDA and PLGA-gelatine surfaces, as shown in Fig. 4. The strategy of using TGF-β1 complexed with BSA as a surface coating—rather than directly incorporating TGF-β1 into the bulk scaffold—was chosen to enhance both bioavailability and functional presentation of the growth factor. Direct incorporation of TGF-β1 within scaffold matrices often leads to initial burst release, and loss of bioactivity due to denaturation during processing, which limits spatial control and sustained signalling. In contrast, BSA acts as a carrier and stabilising agent that protects TGF-β1 active conformation and enables more gradual, surface-mediated presentation to cells.

The TGF-β1/BSA complex interacts with PDA and PLGA-gelatine surfaces primarily via BSA, which prevents direct contact between TGF-β1 and the surfaces. This binding mode suggests that the molecular architecture and surface chemistry of PDA and PLGA-gelatine surfaces influences the orientation and interaction mode of the TGF-β1/BSA complex. The differential BSA binding to each surface may be attributed to the unique and distinct physicochemical properties of PDA and PLGA-gelatine, such as hydrophobicity, surface charge, and functional groups present on the surface. PDA surfaces are known for high hydrophilicity, strong surface charge, and abundant functional groups such as amines and catechols. These properties enhance BSA binding through noncovalent interaction. In addition, PLGA-gelatine surfaces exhibit a different profile with hydrophobicity and a varied distribution of functional groups such as carboxyl, amino, and hydroxyl groups. The PLGA is relatively hydrophobic, while gelatine introduces hydrophilic and functional groups, which can potentially interact with BSA. The combination of noncovalent interactions enables BSA to bind effectively to the PLGA-gelatine surface. Alongside, it demonstrates that BSA can facilitate the indirect interaction of TGF-β1 with the surface, enhancing TGF-β1 availability to cells through this complexation. This complexation likely promotes a more controlled release and presentation of TGF-β1 to cells, thereby enhancing its biological activity. However, these findings necessitate further investigation to confirm the biological implications of this complexation. We have conducted additional cellular experiments to validate the impact of BSA-mediated TGF-β1 surface interactions on cellular responses, which are critical for understanding the potential therapeutic benefits of this approach.

### 3.6 Cell-scaffold interactions

**Fig. 5:**
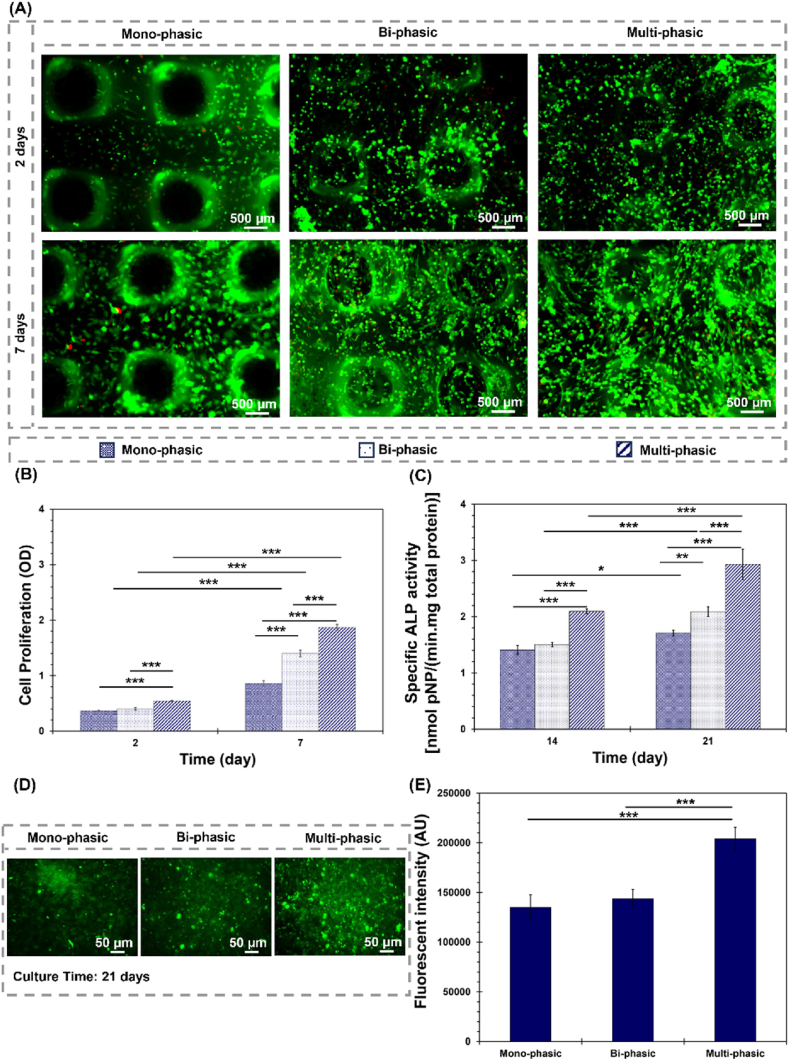
Cell-scaffolds interactions. (A) Fluorescence staining of bone marrow mesenchymal stem cells using calcein-AM and propidium iodide after 2 and 7 days of culture on mono-phasic (subchondral bone-like layer), bi-phasic (on electrospun cartilage-like layer), and multi-phasic scaffolds. (B) Proliferation rates of bone marrow mesenchymal stem cells on scaffolds over the incubation period (2 and 7 days), (∗∗∗P < 0.001). (C) ALP activity in bone marrow mesenchymal stem cells cultured on the scaffolds for 14 and 21 days, (∗P ≤ 0.05; ∗∗P < 0.01; ∗∗∗P < 0.001). (D, E) Fluorescence imaging and quantitative analysis of hydroxyapatite deposition on the scaffolds, assessed by the OsteoImage assay after 21 days of cell culture, (∗∗∗P < 0.001).

The study conducted a comprehensive investigation into the dynamic interplay between cells (sBMCs, human osteoblasts, and human chondrocytes) and scaffolds, revealing intricate interactions. The results from Calcein AM staining (Fig. 5(A)) performed at specific time points (2- and 7-days post-culture) affirmed the scaffolds' significant role as anchorage sites for cells. This anchorage capability and further cell viability was notably enhanced by coating the scaffolds with TGF-β1/BSA. After 2 days in culture, clusters of viable cells began to appear, and by day 7, cell density had noticeably increased. Here, the porous and hydrophilic microstructures of the scaffolds played a crucial role in facilitating oxygenation and fluid transfer, which are essential for sustaining cell viability and functionality [91]. The observation of viable cells within the scaffold matrix after a short period is consistent with previous studies highlighting the importance of the scaffold architecture and surface properties in supporting cell attachment, viability, and proliferation [92]. Furthermore, the observed enhancement in cell viability over time suggests that the scaffold microenvironment provides conducive conditions for cell growth and function. In the viability experiment, bi-phasic scaffolds exhibited a higher density of viable cells compared to mono-phasic scaffolds, indicating the effectiveness of the electrospun layer in promoting cell adhesion and viability. Notably, multi-phasic scaffolds with a TGF-β1/BSA coating demonstrated the highest density of viable cells. This finding aligns with the role of growth factor (TGF-β1) in up-regulating cell behaviour and tissue regeneration [93]. Also, protein (TGF-β1/BSA) deposition significantly impacted the scaffold's surface stability and integrity, establishing a beneficial environment conductive to cell survival [94]. These findings highlight the significance of protein (TGF-β1/BSA) coating in offering specific binding sites for cell receptors or integrins, thereby promoting strong connections with the scaffold's surface [22].

The assessment of cellular viability and proliferation, as depicted in Fig. 5(B), has revealed dynamic cellular activity over time. Initially, there was a low number of viable cells. However, a marked increase in cell viability was observed across all experimental groups by the 7th day of incubation. This effect underscores a cellular response to the scaffold's chemical composition and architecture, thereby indicating their potential for promising cytocompatibility and sustained cell proliferation over extended incubation durations. The observed cell proliferation on mono-phasic scaffolds over the incubation period may be attributed to surface modification with PDA. The incorporation of PDA enhances scaffolds hydrophilicity and bio-adhesiveness, thereby fostering a conducive microenvironment for cell attachment and proliferation, as similarly observed by Xu et al. [95]. It is noteworthy that while no significant disparities were noted between mono-phasic and bi-phasic scaffolds at day 2, a marked augmentation in cell proliferation became evident after 7 days of culture. This delayed response in bi-phasic scaffolds could plausibly be influenced by factors such as the presence of silicon ions [68] within electrospun layer, indicating the nuanced interplay between scaffolds composition and cellular behaviour. Moreover, TGF-β1/BSA-coated scaffolds (multi-phasic scaffolds) showed amplified cell proliferation compared to bi-phasic scaffolds in both 2 and 7 days culture periods. Here, the negative charge provided by the protein complex promotes electrostatic interactions with positively charged cell surfaces. Also, the hydrophilic nature of TGF-β1/BSA on the surface further enhances cell proliferation.

The enzymatic activity of ALP, a crucial regulator in bone mineralization and a widely used biomarker for osteogenesis [[96], [97], [98]], was evaluated at the 14-day and 21-day intervals post-cell culture. Spec. ALP activity showed significant augmentation across all experimental groups by the end of the 21-day period (Fig. 5(C)). This increase, particularly evident in the multi-phasic scaffolds, indicates enhanced osteogenic activity within this group. This observation aligns with prior research indicating the pivotal role of spec. ALP activity as an indicator of osteogenesis [99]. The quantification and qualitative assessment of mineralization using the Lonza OsteoImage kit at the 21-day post-cell culture (Fig. 5(D and E)) demonstrated mineralization capability across all scaffolds. Notably, the multi-phasic scaffolds exhibited a significantly greater capacity to induce hydroxyapatite mineralization compared to the other experimental groups, whereas no significant difference was observed between the mono-phasic and bi-phasic constructs. This heightened effect is plausibly ascribed to the superior bioactivity inherent in the multi-phasic scaffolds. The intricate design and chemistry of multi-phasic scaffolds likely creates an environment conducive to mineralization processes, fostering enhanced osteogenic outcomes [100]. Moreover, it is plausible that the presence of TGF-β1 within the multi-phasic constructs compared with mono-phasic and bi-phasic ones contributes as well as its synergistic cooperation with PDA and simvastatin to this phenomenon. TGF-β1 is known to exert regulatory effects on the expression of key matrix proteins or enzymes essential for facilitating mineralization processes [101].

**Fig. 6:**
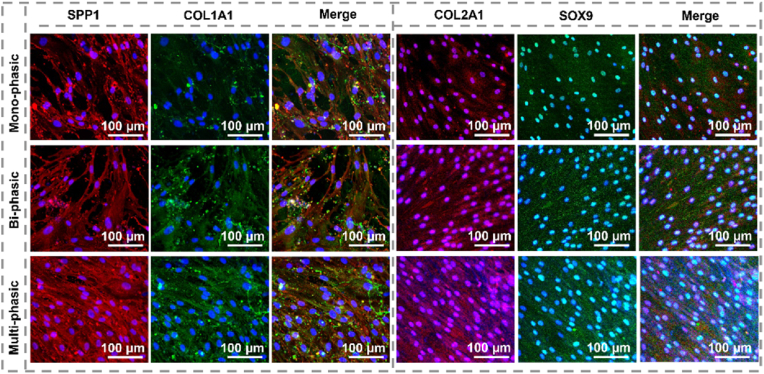
Osteogenic and chondrogenic differentiation of human osteoblasts and human chondrocytes. Immunofluorescence staining of SPP1 and COL1A1 in human osteoblasts, and SOX9 and COL2A1 in human chondrocytes, following 21 days of culture on mono-phasic (subchondral bone-like layer), bi-phasic (on electrospun cartilage-like layer), and multi-phasic scaffolds.

The influence of TGF-β1/BSA surface modification on chondrogenic and osteogenic differentiation was investigated through immunocytochemistry analysis, as illustrated in Fig. 6. The analysis cantered on assessing the expression levels of SPP1 and COL1A1 following a 21-day culture period of human osteoblasts, as well as the expression of SOX9 and COL2A1 after a comparable duration of human chondrocyte culture, employing immunofluorescence staining techniques. Here, it is expected scaffolds provide biochemical and topographical cues that guide tissue-specific differentiation, making it suitable for both direct cell-seeding or stem cell-based approaches. While MSC-based strategies are highly relevant for translational applications, they introduce additional biochemical variables. The integration of human osteoblasts and chondrocytes into this study was undertaken with the objective of enhancing the biological significance. This approach was chosen due to its capacity to closely mimic human physiology and ensure direct evaluation of scaffold's bioactivity. Unlike MSC, which require extrinsic differentiation cues, chondrocytes and osteoblasts exhibit inherent tissue-specific phenotypes, making them ideal for assessing whether the scaffolds themselves support the maintenance of native cartilage- and bone-like microenvironments. The results demonstrated a significant augmentation in the expression of SOX9 and COL2A1, pivotal markers indicative of chondrogenic differentiation, within scaffolds incorporating TGF-β1/BSA coatings. SOX9, a pivotal transcription factor, assumes a fundamental role in regulating chondrogenesis, exerting upstream control over various genes involved in cartilage formation [102]. Meanwhile, COL2A1, a major component of the ECM in cartilaginous tissues, contributes to structural integrity and functional properties of cartilage, thus serving as a reliable marker for chondrocyte differentiation [103]. Furthermore, the observed upregulation of SPP1 and COL1A1 expression in response to TGF-β1/BSA-coated scaffolds underscore their potential in promoting osteogenic differentiation. SPP1, recognized for its multifunctional properties, exerts substantial influence on the regulation of mineralization processes, a phenomenon well-documented in prior research [104]. Additionally, COL1A1, serving as a principal constituent of the bone matrix, contributes to structural integrity, aids mineralization, and modulates cellular signalling pathways, thereby playing crucial roles in bone formation [105].

These findings collectively indicate that integrating TGF-β1/BSA coatings into multi-phasic scaffolds enhances the expression of critical markers associated with both osteogenic and chondrogenic differentiation pathways. In contrast, both mono-phasic and bi-phasic scaffolds exhibited diminished expression levels of key genes (SPP1, COL1A1, SOX9, and COL2A1), suggesting a lower capacity for osteogenic and chondrogenic differentiation. Such outcomes underscore the potential of biomimetic coatings in orchestrating favourable cellular responses conducive to osteochondral tissue repair and regeneration. In multi-phasic scaffolds, the incorporation of PDA create a conducive environment for osteoblast functionality and differentiation [106]. The surface properties of PDA further facilitate the absorption of bioactive molecules, thereby enhancing cellular differentiation and promoting an osteoinductive environment. Simvastatin has shown the ability to enhance osteoblast activity crucial for bone formation [107]. Moreover, simvastatin enhances mineralization on PDA surfaces, indicating its potential to improve bone strength and density. Importantly, coatings of TGF-β1/BSA complex have shown significant enhancement in cell adhesion, proliferation, and osteogenic-chondrogenic differentiation, owing to their ability to efficiently absorb bioactive molecules, thereby enhancing solubility and bioavailability. Specifically, TGF-β1 plays a pivotal role due to its regulatory influence on various cellular processes. Activation of TGF-β1 signalling pathways upon interaction with cells on the scaffolds surface may lead to the secretion of ECM components crucial for osteochondral regeneration. TGF-β1 is known to regulate bone and cartilage tissue formation, exerting effects on osteogenic and chondrogenic differentiation, which are essential processes in bone and cartilage formation [108,109]. Additionally, applying BSA coating could mitigate nonspecific protein adsorption, promoting a favourable surface for cell interaction [110]. The collaborative interplay among these factors, particularly proteins, augments cell-scaffold interactions, stabilizes scaffold surfaces, and enables delivery of bioactive molecules. Consequently, this interplay fosters cell adhesion, proliferation, and the expression of chondrogenic and osteogenic genes. Investigating deeper these complex interactions holds promise for garnering significant insights applicable to translational endeavours within osteochondral regenerative medicine.

## 4 Conclusion

This study represents an advancement in the design and fabrication of scaffolds with sufficient complexity for the reconstruction of damaged anisotropic osteochondral tissue. Through the engineering of hierarchical scaffolds and the integration of state-of-the-art techniques, a customized multi-phasic scaffold architecture was developed. To achieve this, a subchondral bone-like layer was constructed using a bioinspired approach involving PDA functionalization and simvastatin loading onto PLGA scaffolds, which were produced using an environmentally friendly approach via 3D printing. Subsequently, a layer of PLGA-gelatine electrospun fibres covered the scaffold surface to mimic cartilage-like structure. A customized multi-phasic scaffolds layout was put into action, modifying bi-phasic scaffolds by incorporating a TGF-β1/BSA complex, aiming for synergistic effects to boost osteogenic and chondrogenic potential. The comparison of three experimental groups revealed the distinct biological contribution of each material design layer, enabling a stepwise understanding of how architectural and biochemical modifications enhance the osteochondral regeneration potential.

The concentrations of PDA, simvastatin, TGF-β1/NSA were selected based on previously published studies demonstrating their biological efficacy in promoting osteogenic and chondrogenic responses. The aim of the current study was not to optimize or quantify the release kinetics of individual components in isolation, but rather to evaluate the biological impact and feasibility of integrating multiple biofunctional strategies within a unified, stratified scaffold system. By focusing on the synergistic performance of the multi-phasic scaffold as a whole, this study lays the foundation for future work where release kinetics can be systematically evaluated in the context of preclinical validation.

In this study, comprehensive morphological and physicochemical evaluations, complemented by protein interaction simulations and *in-vitro* assessments, collectively confirmed the effectiveness of the scaffolds. The fabrication of columnar pores within the 3D printed layer of stratified scaffolds, along with the incorporation of electrospun fibres, should enable optimized nutrient transport and ion exchange, establishing an environment conducive to cellular activities. The biomimetic multi-phasic scaffolds exhibited favourable adhesion, viability, and proliferation of sBMSCs, indicating their cytocompatibility. Additionally, the protein interaction simulations indicated that inclusion of TGF-β1/BSA complex on the scaffolds can further enhance TGF-β1 accessibility to cells. Furthermore, the upregulation of ALP expression and the observed potential for the deposition of hydroxyapatite nodules by cells, in conjunction with increased expression levels of osteogenic markers (SPP1 and COL1A1), underscored the scaffolds' osteogenic performance for osteochondral tissue reconstruction. Additionally, the expression of chondrogenic markers (SOX-9 and COL2A1) confirmed the chondrogenic potential of the multi-phasic scaffolds for osteochondral tissue regeneration. While this study demonstrates the promising *in-vitro* performance of the multi-phasic scaffold, there remains a clear need for comprehensive characterization of its mechanical properties, particularly interfacial strength. This should be complemented by long-term biological evaluation to assess the structural integrity and functional performance of the engineered tissues over time. Future work should focus on *in-vivo* validation using clinically relevant osteochondral defect models to investigate tissue integration, immune response, and mechanical functionality under physiological conditions. Furthermore, although the scaffold exhibited controlled degradation over a 4-week period, its long-term degradation and remodelling dynamics in an *in-vivo* environment remain to be fully characterized.

The stratified scaffold system was developed with a focus on future translation. Herein, the scaffold could be applied either as an off-the-shelf or custom press-fit implant, conforming precisely to the geometry and depth of the osteochondral defect. The 3D-printed subchondral bone-like layer offers structural integrity, while the electrospun, nanofibrous cartilage-like layer provides a favourable microenvironment for chondrogenic differentiation. The protein coating strategy (e.g., TGF-β1/BSA) further enhances the scaffold's biological performance and reduces the need for external growth factor supplementation, simplifying intraoperative handling. Moreover, the application of additive manufacturing technologies enables the fabrication of individualised, patient-specific scaffolds, precisely tailored to anatomical contours and defect dimensions. This personalized approach has the potential to improve implant-host interface integration, minimise intraoperative modification, and enhance functional repair outcomes.

Thus, by amalgamating advanced materials engineering with biomimetic design principles, the innovative scaffold technology presented in this work paves the way for personalized therapeutic interventions, addressing the intricate challenges inherent in osteochondral tissue regeneration. Given the promising results observed in terms of cell viability, gene expression, and scaffold performance, *in-vivo* validation using an osteochondral defect model is a planned next step.

## CRediT authorship contribution statement

**Farnaz Ghorbani:** Writing – review & editing, Writing – original draft, Visualization, Validation, Project administration, Methodology, Investigation, Funding acquisition, Data curation, Conceptualization. **Behafarid Ghalandari:** Writing – review & editing, Visualization, Methodology, Investigation. **Rainer Detsch:** Writing – review & editing, Visualization, Methodology, Investigation. **Chaozong Liu:** Writing – review & editing, Supervision, Project administration, Conceptualization. **Aldo R. Boccaccini:** Writing – review & editing, Supervision, Project administration, Conceptualization.

## Funding

This work was supported by 10.13039/100005156Alexander von Humboldt Foundation (Fellowship to FG).

## Declaration of competing interest

The authors whose names are listed certify that they have NO affiliations with or involvement in any organization or entity with any financial interest (such as honoraria; educational grants; participation in speakers' bureaus; membership, employment, consultancies, stock ownership, or other equity interest; and expert testimony or patent-licensing arrangements), or non-financial interest (such as personal or professional relationships, affiliations, knowledge or beliefs) in the subject matter or materials discussed in this manuscript.

## Data Availability

Data will be made available on request.
